# Combinatorial Effects of Thymoquinone on the Anticancer Activity and Hepatotoxicity of the Prodrug CB 1954

**DOI:** 10.3797/scipharm.1211-15

**Published:** 2013-01-03

**Authors:** Wamidh H. Talib, Majed M. AbuKhader

**Affiliations:** 1 Department of Clinical Pharmacy and Therapeutics, Applied Science University, Amman 11931, Jordan.; 2 Department of Pharmacy, Oman Medical College, Muscat, Sultanate of Oman.

**Keywords:** *Nigella sativa*, CB 1954, Thymoquinone, Liver toxicity, Anticancer drugs

## Abstract

**Background:**

One of the major causes of clinical trial termination is the liver toxicity induced by chemotherapeutic agents. Treatment with anticancer drugs like CB 1954 (5-(Aziridin-1-yl)-2,4-dinitrobenzamide) is associated with significant hepatotoxicity. Thymoquinone (TQ), extracted from *Nigella sativa,* is reported to possess anticancer and hepatoprotective effects. The aims of the present study were to use TQ to reduce hepatotoxicity associated with CB 1954 and to augment its anticancer activity against the resistant mouse mammary gland cell line (66 cl-4-GFP).

**Method:**

Balb/C mice were transplanted with the 66cl-4-GFP cell line and *in vivo* antitumor activity was assessed for CB 1954 (141 mg/kg), TQ (10 mg/kg), and a combination of CB 1954 and TQ. Changes in tumor size and body weight were measured for each treatment. Histological examination of tumors and liver tissue samples was performed using the standard hematoxylin/eosin staining protocol, and serum levels of the liver enzymes AST and ALT were used as biomarkers of hepatotoxicity.

**Results:**

Severe liver damage and elevated plasma levels of AST and ALT were observed in the group treated with CB 1954. Treatment of tumor-bearing mice with a combination of CB 1954 and TQ caused a significant regression in tumor size and induced extensive necrosis in these tumors. The combination also protected the liver from drug-induced damage and reduced the plasma levels of AST and ALT to their normal ranges.

**Conclusion:**

These results suggest that the use of TQ with CB 1954 can reduce CB 1954-induced hepatotoxicity and enhance its anticancer activity, indicating the potential use of this combination in clinical studies.

## Introduction

Conversion of a normal cell into a cancer cell is a multi-step process including the induction of instability in genes, abnormal gene expression, angiogenesis, metastasis, and immune evasion [1]. Thus, using a single agent to target all of these pathways may fail to provide an efficient treatment for cancer. Accordingly, different combinations were prepared and tested against different cancers. Due to their limited toxicity, natural products were a preferred choice in preparing such combinations. A combination of phytochemicals extracted from spices including capsaicin, trans-anethole, thymoquinone, diosgenin, and allicin exhibited high potential to suppress different pathways in carcinogenesis such as proliferation, signal transduction, apoptosis evasion, and angiogenesis [2]. Some of the cancer induction pathways were also inhibited by different polyphenols present in honey such as galangin, quercetin, kaempferol, acacetin, pinocembrin, pinobanksin, and apigenin [3]. In addition to their potential of targeting different cancers, natural products may also participate in reducing the toxicity of commercially available anticancer agents [4].

One of the major reasons of clinical trial termination is drug-induced toxicity [5]. Recent estimates showed that liver failure induced by drugs accounts for 50% of the total acute liver failure reported in the USA [6].

CB 1954 (5-(Aziridin-1-yl)-2,4-dinitrobenzamide) is an alkylating agent which can be activated by some reductases to produce a toxic form that has the ability to induce cell death through the induction of interstrand DNA cross-links [7]. Tumors that showed resistance to anti-neoplastic agents are susceptible to cell death using activated CB 1954 [8]. CB 1954 is currently under study as a prodrug in combination with enzymes such as *Escherichia coli* B nitroreductase and NADP(H) quinone oxidoreductase [6].

Initial clinical toxicity studies on CB 1954 revealed dose-limiting diarrhea and liver toxicity [7]. Further studies reported temporal transaminitis in some patients [9, 10].

Thymoquinone (TQ) is a naturally-occurring volatile oil extracted from its black seeds (*Nigella sativa*). Previous studies on TQ showed *in vitro* and *in vivo* anticancer, anti-inflammatory, and anti-oxidant activities [11]. The combination of TQ with other natural compounds like diosgenin exhibited antineoplastic activity against squamous cell carcinoma *in vitro* and sarcoma-180-induced tumors *in vivo*[12]. The effect of TQ as an immunomodulator was also reported [13].

Toxicity studies showed that TQ can reduce the hepatotoxicity induced by some compounds including carbon tetrachloride [14], *tert*-butyl hydroperoxide [15], and cyclophosphamide [4]. Furthermore, TQ had no effect on liver integrity and hepatic enzyme activity when tested on mice and rats [16].

Importantly, the intraperitoneal therapeutic dose of TQ is 10–15 times lower than its LD_50_ value [17]. Thus, it is reasonable to assume that TQ is a safe compound and has the potential to work as a therapeutic and hepatoprotective agent in combination with other biologically active, but relatively more toxic compounds. The present study was conducted mainly to test the potential of TQ to reduce the hepatotoxicity associated with the anticancer prodrug CB 1954 and to evaluate the possible improvement in its anticancer activity.

## Materials and methods

### Chemicals

Thymoquinone (2-isopropyl-5-methyl-1,4-benzoquinone) and CB 1954 (5-(Aziridin-1-yl)-2,4-dinitrobenzamide) were purchased from Sigma Chemical Co. (St. Louis, MO. USA).

### Tumor cell line and culture conditions

The mouse mammary cancer cell line (66CL-4-GFP) was kindly provided by Dr. Bob Sanders (Department of Genetics and Microbiology, University of Texas, Austin, USA). The cell line was derived from a spontaneous mammary tumor in Balb/C mice and isolated as the 6-thioguanine resistant clone. These cells were transfected with green florescence protein (GFP). Cells were maintained using DMEM-F12 supplemented with 10 % FBS, 29 μg/ml L-glutamine, 40 μg/ml gentamicin and 2.4 mg/ml HEPES buffer.

### Experimental animals

Six-to-eight-week-old female Balb/C mice were used in this study. Mice were kept in separate cages with wooden shavings as bedding. The environmental parameters were: temperature around 25 °C, 50–60% humidity, and continuous air ventilation. The research followed the international ethical standards for the Care and Use of Laboratory Animals.

### Tumor inoculation

The mouse mammary tumor cells (66cl-4-GFP) were harvested by trypsinization, centrifuged, washed, and re-suspended in MEM-F12 media at a density of 1 X 10^6^/100 μl. Cell viability was assessed the using trypan blue exclusion method. Mice (six weeks old, 20–25 g weight) were injected subcutaneously in the abdominal area using a 23-gauge needle syringe with 2 × 10^6^ cells (for each one) suspended in 200 μl phosphate buffer saline (PBS).

### Selection of TQ and CB 1954 doses

The concentration of TQ used in this study to treat mice was 10 mg/kg/day. This concentration was selected because it showed no toxicity since it is ten times lower than the reported LD_50_ value (104.7 mg/Kg/day) of TQ [17]. This concentration also showed good protective activity against different toxic agents as indicated by previous studies [18, 19]. For CB 1954, the concentration was 141 mg/kg. This concentration was selected because it is associated with liver damage and transaminitis in mice [6].

### Antitumor activity testing

Tumor-bearing mice (N=6) were placed in four groups so that the average tumor volume for all groups was closely matched. Treatments began 14 days following tumor cell inoculation. Group 1 served as a negative control and received daily intraperitoneal injections (200 μl) of the vehicle (olive oil). Group 2 was exposed to daily intraperitoneal injections (200 μl) of TQ dissolved in olive oil for five days at the concentration = 10mg/kg. Six days after the beginning of the treatment, group 3 received a single intraperitoneal injection (200 μl) of CB 1954 dissolved in olive oil at the concentration = 141 mg/kg. Group 4 received a combined treatment consisting of the intraperitoneal injection of TQ (10mg/kg) for the first five days of the treatment followed by a single intraperitoneal injection of CB 1954 (141 mg/kg) at day six of the treatment. Mice were monitored during the eight days of the treatment period and the tumor size was measured every two days using the equation: length × width^2^ × 0.5. At day eight of the treatment, blood samples were collected from all mice for biochemical tests, and tumor-bearing mice in all groups were sacrificed and their tumors and livers were dissected and stored in 10 % saline formalin for further testing.

The dose (141 mg/kg) of CB 1954 was selected to induce hepatotoxicity and transaminitis as indicated in a previous study [6]. The hepatoprotective effect of TQ was tested through the pretreatment of mice with (10mg/kg/day) TQ for five days [20].

### Assessment of liver function

Alanine transaminase (ALT) and aspartate transaminase (AST) activities were determined colorimetrically as previously described [20] using commercially available kits (Linear chemicals, Barcelona, Spain).

### Histological examination of tumor and liver sections

Dissected tumors and livers (5 × 5 × 4 mm) fixed in 10 % saline formalin were gradually dehydrated using serial ethanol concentrations of 80 %, 95 %, and 100 %. Dehydrated organs were cleared two times using xylene (2 h each). Infiltration was performed by exposing organs to wax two times for 90 minutes each. Dehydration, clearing, and infiltration were performed using a tissue processor (Thermo Shandon, UK). Paraffin sections (4 μm thick) were prepared using a rotary microtome (Reichert, Germany). Sections were attached to clean slides and the standard hematoxylin-eosin procedure was used to stain tumor sections for histological examination.

### Statistical analysis

The results were expressed as the mean ± standard deviation (SD). Variation between different treatments was measured by one-way analysis of variance (ANOVA) followed by the *t*-test to evaluate the significant differences between the groups. Significance was set at *P* ≤ 0.05.

## Results

Treatment of tumor-bearing mice with 10 mg/kg TQ showed significant (P< 0.05) ability to reduce tumor growth with a percentage change in tumor size of (−1.25%) compared with that of the untreated mice (+ 209.82%) (Table 1). A greater reduction in tumor growth was observed in tumor-bearing mice treated with 141 mg/kg CB 1954 with a percentage change in tumor size of (−10.34%). The highest reduction in tumor size was recorded for mice treated with a combination of TQ and CB 1954 (Table 1) with a decrease in tumor size of (−21.58). Measuring the change in body weight showed a decrease in body weight for all treatments compared with the control. The highest reduction in body weight was observed in mice treated with a combination of TQ and CB 1954 with a percentage change in body weight of (−14.09%) compared with (+ 4.16) recorded for untreated mice (Table 1). A slight (−1.68) decrease in body weight was observed in mice treated with TQ, while mice treated with CB 1954 showed a reduction in body weight of (−9.01) (Table 1).

**Fig. 1:**
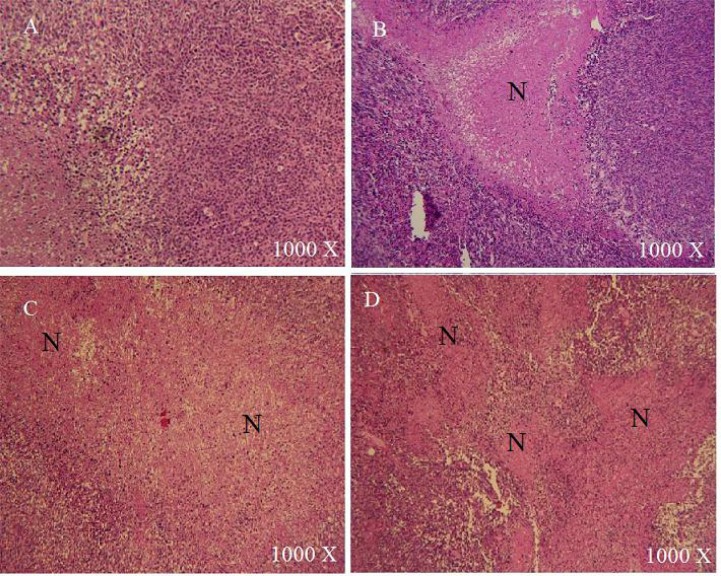
Hematoxylin/eosin staining of tumors treated with vehicle (A), 10mg/kg TQ (B), 141 mg/kg CB1954(C), and a combination of TQ and CB1954 (D). N: necrotic area. Extensive necrosis was evident in tumors treated with a combination of TQ and CB1954 (D).

In order to gain more insight into the effect of each treatment, tumors of similar sizes from all groups were sectioned and stained using the standard Hematoxylin/eosin stain. Large necrotic areas were observed in tumors treated with TQ compared with the control group, where limited or no necrosis was detected (Fig. 1). The necrotic regions in tumors treated with CB 1954 were larger than those observed in the TQ group. The combination of TQ and CB 1954 resulted in extensive necrosis with necrotic regions larger and more frequent than other treatments (Fig. 1).

**Fig. 2:**
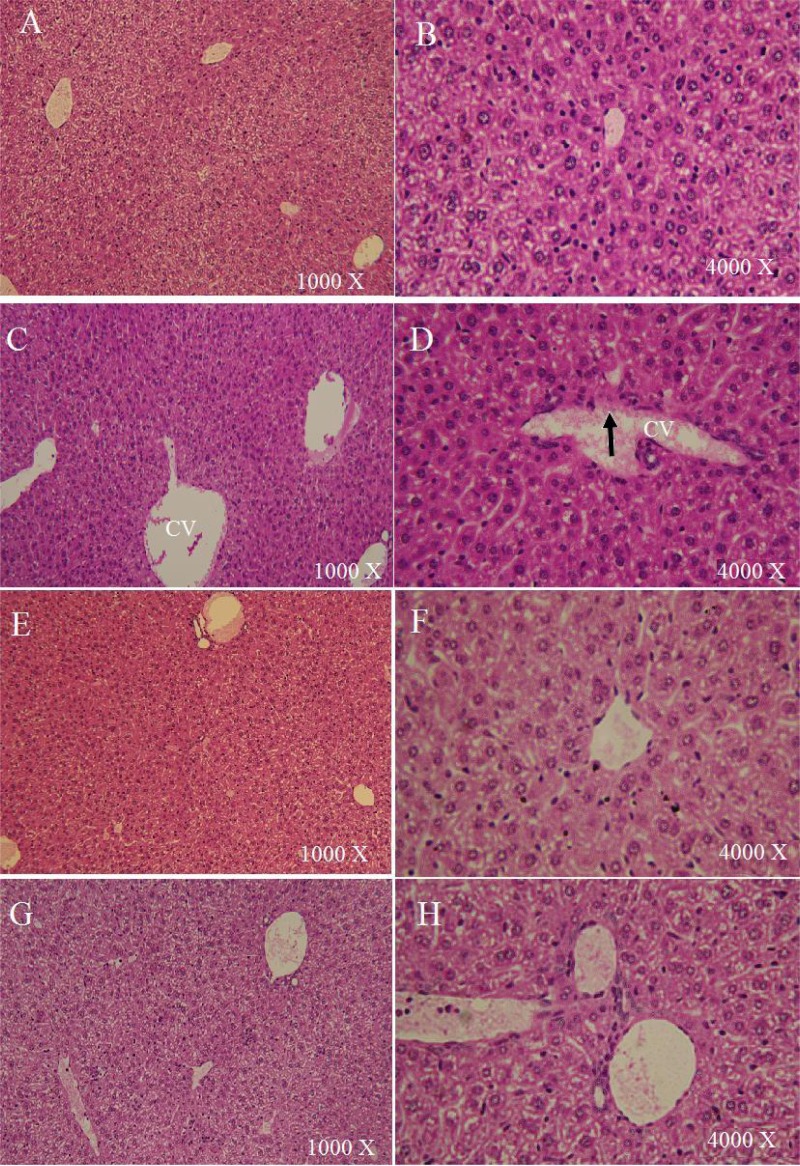
Hematoxylin/eosin staining of livers treated with vehicle (A, B), 141 mg/kg CB1954(C,D), 10mg/kg TQ (E, F), and a combination of TQ and CB1954 (G, H). Endothelium detachment (arrow) and sinusoid dilation were observed in livers treated with 141 mg/kg CB1954 (C, D). Other treatments showed no histological damage.

The effect of TQ on the CB 1954-induced hepatotoxicity was examined using histopathological changes in liver sections as well as the plasma levels of ALT and AST. Histopathology results revealed that animals treated with TQ showed well-preserved liver histology similar to the histology of the control group with normal central veins, sinusoids, and hepatocytes (Fig. 2). On the contrary, livers from the group treated with CB 1954 showed detached endothelia in the central veins, dilated sinusoids, and infiltration of inflammatory cells (Fig. 2). The histological changes associated with CB 1954 toxicity were modulated by TQ pretreatment which resulted in livers with normal central veins, limited sinusoidal dilation, and less infiltration of inflammatory cells.

In order to correlate between the histopathological results and plasma biomarkers of hepatotoxicity, plasma levels of AST and ALT were measured for all treatments. As shown in Table 2, treatment of mice with CB 1954 caused a significant (P< 0.05) increase in AST and ALT levels from 22.24 and 8.03 to 57.28 and 22.91 IU/L, respectively. A slight increase in AST and ALT levels was observed in mice treated with TQ with AST and ALT values of 26.59 and 11.82 IU/L, respectively. Although mice treated with a combination of TQ and CB 1954 showed an increase in AST and ALT levels (24.48 and 15.77 IU/L, respectively), this increase is limited compared with the values obtained in the group treated with CB 1954 alone (57.28 and 22.91 IU/L, respectively).

## Discussion

Although CB 1954 is considered to be a promising agent in cancer therapy, several phase I trials reported the hepatotoxic effects of this compound mostly in the form of transaminitis [6].

Thymoquinone extracted from *Nigella sativa* oil has been studied for its anticancer, anti-inflammatory, anti-oxidant, and hepatoprotective activities [2, 11]. Accordingly, this study was designed to test the hypothesis that TQ may reduce hepatotoxicity and enhance the anticancer activity of CB 1954.

Although previous studies have shown that TQ has the potential to selectively inhibit different cancers, including prostate cancer [21], fibrosarcoma [22], myeloblastic leukemia [23], colorectal carcinoma [24], and breast adenocarcinoma [25], our results showed for the first time that TQ has the potential to also target 6-thioguanine-resistant mouse mammary cancer. We also found that CB 1954 anticancer activity was potentiated in combination with TQ. Such results is in accordance with previous findings that reported the ability of TQ to enhance the effect of therapeutic agents like doxorubicin and 5- fluorouracil [25, 26], in addition to Cisplatin [21].

Previous studies reported the ability of TQ to induce the expression of some enzymes including quinone reductase [28]. Interestingly, CB 1954 is one of the substrates for quinone reductase [29]. Thus, it is reasonable to assume that one mechanism underlying the anticancer effect of this combination could be attributed to the over-expression of quinone reductase in cancer cells, which reduces CB 1954 converting it into its active form. Our results showed that the tumor cell death occurs mainly in the core of the tumor where the oxygen concentration is low and cancer cells are more resistant to chemotherapeutic agents. This result is supported by the previous findings that reported high CB 1954 reduction under anaerobic conditions [30]. A recent study showed that one of the anticancer effects of TQ is exerted by inhibiting angiogenesis [31]. Angiogenesis inhibition creates more hypoxic regions in the tumor tissue and this will facilitate the conversion of CB 1954 into its therapeutic form. This may explain the results obtained in our study where more cell death was observed in tumors treated with a combination of TQ and CB 1954.

Hepatotoxicity and/or nephrotoxicity are usually associated with toxicity related to anticancer drugs. The results of this study showed that the treatment of mice with CB 1954 is associated with the dysregulation of liver histology and function as indicated by the abnormal histology of the liver and the increase in plasma levels of ALT and AST. These results agree with previous studies that reported elevated levels of AST and ALT in addition to liver damage in mice treated with CB 1954 [6]. However, these toxicity effects were ameliorated in mice treated with a combination of TQ and CB 1954, indicating the ability of TQ to protect against CB 1954-induced toxicity. Our results are consistent with previous studies that reported the ameliorative effects of TQ against toxicity of different compounds including cisplatin [32], cyclophosphamide [2], and carbon tetrachloride [14].

## Conclusion

The use of TQ with CB 1954 can reduce CB 1954-induced hepatotoxicity and enhance its anticancer activity, indicating a potential use of this combination in clinical studies. However, further studies are needed to understand the detailed mechanism of action of this combination and the range of cancers that may respond to this combination therapy.

## Figures and Tables

**Tab. 1:** Effects of different treatments on mice weights and tumor sizes

**Treatment**	**body weight (g)**			**tumor size (mm^3^) ± SEM**		

	**initial**	**final**	**% change**	**initial**	**final**	**% change**
Negative control	23.10	24.06	+4.16	55.90 ± 0.14	173.19 ± 2.88	+209.82
TQ	22.60	22.22	−1. 68	72.60 ± 0.45	71.69 ± 3.12	−1.25
CB1954	23.30	21.20	−9.01	79.02 ± 0.20	70.85 ± 3.91	−10.34
TQ + CB1954	23.50	20.19	−14.09	71.22 ± 0.31	55.85 ± 3.20	−21.58

% change in weight = (final weight − initial weight)/initial weight × 100;

% change in tumor size = (final size − initial size)/initial size × 100.

**Tab. 2:** Effect of different treatments in plasma levels of liver enzymes.

**Treatment**	**AST (IU/L) ± SEM**	**ALT(IU/L) ± SEM**
Vehicle	22.24 ± 0.52	8.03 ± 2.19
TQ	26.59 ± 2.78	11.82 ± 1.64
CB1954	57.28 ± 5.22	22.91 ± 0.76
TQ + CB1954	24.48 ± 4.07	15.77 ± 2.37

## Authors' Statements

### Animals Rights

The study followed the institutional and international Guide for the Care and Use of Laboratory Animals.
